# Identification of Candidate Genes Related to Inflammatory Bowel Disease Using Minimum Redundancy Maximum Relevance, Incremental Feature Selection, and the Shortest-Path Approach

**DOI:** 10.1155/2017/5741948

**Published:** 2017-02-14

**Authors:** Fei Yuan, Yu-Hang Zhang, Xiang-Yin Kong, Yu-Dong Cai

**Affiliations:** ^1^Department of Science & Technology, Binzhou Medical University Hospital, Binzhou 256603, Shandong, China; ^2^Institute of Health Sciences, Shanghai Institutes for Biological Sciences, Chinese Academy of Sciences, Shanghai 200031, China; ^3^School of Life Sciences, Shanghai University, Shanghai 200444, China

## Abstract

Identification of disease genes is a hot topic in biomedicine and genomics. However, it is a challenging problem because of the complexity of diseases. Inflammatory bowel disease (IBD) is an idiopathic disease caused by a dysregulated immune response to host intestinal microflora. It has been proven to be associated with the development of intestinal malignancies. Although the specific pathological characteristics and genetic background of IBD have been partially revealed, it is still an overdetermined disease and the blueprint of all genetic variants still needs to be improved. In this study, a novel computational method was built to identify genes related to IBD. Samples from two subtypes of IBD (ulcerative colitis and Crohn's disease) and normal samples were employed. By analyzing the gene expression profiles of these samples using minimum redundancy maximum relevance and incremental feature selection, 21 genes were obtained that could effectively distinguish samples from the two subtypes of IBD and the normal samples. Then, the shortest-path approach was used to search for an additional 20 genes in a large network constructed using protein-protein interactions based on the above-mentioned 21 genes. Analyses of the 41 genes obtained indicate that they are closely associated with this disease.

## 1. Introduction

Inflammatory bowel disease (IBD) is a common systemic disease that involves the intestinal tissue [1]. It usually refers to chronic conditions that lead to intestinal inflammation and lesions. With the gradual development of inflammation, the intestinal walls become swollen, inflamed, and ulcerogenic [2]. Due to such lesions, several classical symptoms have been considered to be diagnostic indicators. Abdominal pain or cramping, diarrhea multiple times per day, and bloody stools are all classical symptoms of IBD [3]. Such severe symptoms are induced by violent unhealthy inflammation reactions and lesions in the intestinal tissue. Additionally, several complications outside the digestive tract may also be induced by IBD. Mouth sores and skin problems have both been reported in IBD patients [4]. Furthermore, arthritis is also related to IBD, as well as eye problems [5, 6].

As we have mentioned above, several complications have been identified in IBD patients. Such severe complications and related chronic characteristics strongly increase the risk of death [4–6]. In 2013 alone, thousands of people in the world died from IBD [7]. Additionally, IBD has been proven to be associated with colorectal cancer, with a high mortality. Apart from the risk of death, IBD is a lifetime disease, and life with IBD can be quite challenging. The complications associated with IBD and disease relapse severely impact the quality of life [8]. Therefore, the prevention, diagnosis, and treatment of IBD are quite crucial. It is known that IBD is a widespread disease that can develop at any stage of life. However, the disease usually initiates during the teenage years or the early adulthood of the patients [8]. As we mentioned above, genetic factors participate in the initiation and progression of IBD [9, 10]. Therefore, people with a family history of IBD are at least ten times more likely to suffer from it. Racial factors also contribute to the morbidity of IBD [11].

Although IBD is a very severe and widespread disease, the essential mechanism behind the disease has not been demonstrated clearly. Most people believe that some types of exogenous materials trigger the initiation of inflammation [12, 13]. However, genetic factors may also contribute to the progression of such disease. Several specific genes have been linked to IBD. Pathogenic genes such as IL23R and IL12B play a crucial role in the intestinal immune system, which may induce the initiation of IBD [14, 15]. Several transcriptional factors also contribute to disease progression. The transcription factor NKX2-3 regulates the correct localization of lymphocytes and may further contribute to the immune response in intestinal tissue that induces IBD [16]. Several genes such as ZNF365 and PTGER4 show diversity in different subtypes of IBD and contribute to IBD through their respective methods and pathways [17, 18].

As mentioned above, IBD has several subtypes. Basically, there are two main clinical classifications of IBD: Crohn's disease and ulcerative colitis [17]. Both classifications share the basic symptoms of IBD. However, Crohn's disease can occur anywhere along the digestive tract and typically appears as “skip lesions” between healthy areas [19], while the other type, ulcerative colitis, only involves the colon and rectum. Inflammation and ulcers typically affect only the innermost lining in these areas, with more superficial lesions than those with Crohn's disease [20]. Apart from the differences in clinical symptoms, genetic diversity is also observed between Crohn's disease and ulcerative colitis. Although they share most of the disease-causing genes, genes like ATG16L1, PTGER4, IRGM, and NOD2 have been proven to be specifically related to Crohn's disease but independent with ulcerative colitis [18, 21–23]. The roles of genes such as SLC22A5, ZNF365, and PTPN2 in ulcerative colitis are still unclear, even though they have been proven to be strongly related to Crohn's disease [24, 25].

Because genetic factors have been shown to be related to IBD and its specific subtypes, we developed a new computational method to screen differential expressing genes among different clusters based on a database for Crohn's disease and ulcerative colitis. From the Gene Expression Omnibus (GEO), we obtained the gene expression profiles (information often used to deduce and understand gene functions) for 59 Crohn's disease, 26 ulcerative colitis, and 42 normal samples. Each sample was represented using the expression levels of 12,754 genes. Two feature selection methods, minimum redundancy maximum relevance (mRMR) and incremental feature selection (IFS) [26], and a basic machine learning algorithm, sequential minimal optimization (SMO) [27, 28], were adopted to analyze the gene expression profiles and extract 21 promising candidate genes that could be used to distinguish the samples from the two subtypes of IBD and the normal samples; that is, they may be related to IBD. Furthermore, based on these 21 genes, the shortest-path (SP) approach was employed to identify additional 20 genes in a network constructed using protein-protein interaction (PPI) information. It was concluded that the 41 (21 + 20) genes obtained are closely associated with IBD and can be used to clearly distinguish healthy people from those who have IBD and to identify the subtypes of IBD.

## 2. Materials and Methods

### 2.1. Dataset

We downloaded the gene expression profiles of 59 Crohn's disease, 26 ulcerative colitis, and 42 normal samples from GEO under accession number GSE3365 [29]. The expression levels of 12,754 genes were measured using an Affymetrix Human Genome U133A Array. The gene expression profiles were quantile normalized. Each sample was represented using the expression levels of 12,754 genes; that is, each sample was encoded into a 12754-D vector. These features/genes were analyzed to identify the genes that can best discriminate the samples from these three different classes.

### 2.2. mRMR Method

It is known that some genes can effectively help us discriminate the samples from the three different classes mentioned in Section 2.1, while others offer few or no contributions. To identify these genes, the mRMR method, proposed by Peng et al. [26], was adopted to analyze the gene expression data. The mRMR method employed two criteria, Max-Relevance and Min-Redundancy, to analyze the features. Using the Max-Relevance criterion, the MaxRel feature list can be obtained, in which features are sorted by measuring the relevance between them and sample class labels. Features with high relevance receive high ranks, whereas those with low relevance receive low ranks. It is clearly seen that the rank of a feature in the MaxRel feature list indicates its single contribution to classification. Furthermore, another list, namely, the mRMR feature list, was created using both Max-Relevance and Min-Redundancy criteria. The rank of a feature in this list is determined using the relevance between it and sample class labels and the redundancies between it and the features listed before it. The MaxRel feature list and mRMR feature list in this study were formulated as follows:

MaxRel features list is
  (1) $$\begin{array}{c} F_{MaxRel}=\left[\;f_{1}^{M},f_{2}^{M},\ldots ,f_{N}^{M}\;\right]; \end{array}$$
mRMR features list is
  (2) $$\begin{array}{c} F_{mRMR}=\left[\;f_{1}^{m},f_{2}^{m},\ldots ,f_{N}^{m}\;\right], \end{array}$$

where *N* represents the total number of features. Many investigators have used the mRMR method to analyze various complicated biological systems [30, 31], and it is deemed to be a useful tool for extracting important information from a complicated system. Readers can refer to Peng et al.'s paper [26] for the detailed procedures and principle of this method.

### 2.3. Prediction Engine

SMO is a type of support vector machine that uses Platt's sequential minimal optimization algorithm to train and optimize the support vector classifier. The kernels can be polynomial or Gaussian [27, 28]. For implementing our method, we employed the classifier SMO implemented in Weka [32] as the prediction engine.

### 2.4. Tenfold Cross-Validation

Tenfold cross-validation [33] is a type of cross-validation method that is widely used to examine the performance of a classifier on a given dataset. The given dataset is randomly and equally divided into ten partitions. Samples in each partition are singled out in turn as the test data, while other samples are used to train the classifier. Compared to the jackknife test [34, 35], another popular cross-validation method, this method involves a lower amount of computational time and always yields similar results. Thus, it was used in this study for evaluating the performance of the current prediction engine.

### 2.5. IFS Method

Using the mRMR method, features/genes were sorted and listed in the MaxRel feature list and mRMR feature list. Because the MaxRel feature list sorted features/genes by only measuring their own contributions to classification, the combination of some features/genes with high ranks in this list is not always an optimal combination for classification. The mRMR feature list is more appropriate for this purpose because it further considers the redundancies between features. The IFS method uses the mRMR feature list and the SMO prediction engine to extract the optimal combination of features/genes as biomarkers. First, according to the mRMR feature list *F*_mRMR_ = [*f*_1_^*m*^, *f*_2_^*m*^,…, *f*_*N*_^*m*^], we constructed *N* feature set, denoted by *F*_1_, *F*_2_,…, *F*_*N*_, where *F*_*i*_ = {*f*_1_^*m*^, *f*_2_^*m*^,…, *f*_*i*_^*m*^}; that is, *F*_*i*_ contained the top *i* features in the mRMR feature list. Second, for each *F*_*i*_, SMO was executed on the dataset, in which samples were represented using features in *F*_*i*_, with its performance evaluated by tenfold cross-validation. Finally, we counted the total prediction accuracy and accuracies for each class. The feature set yielding the highest total prediction accuracy was deemed to be the optimal gene set (*G*_optimal_) for IBD, as features in this set may be significant for IBD.

### 2.6. Network Construction from PPI Information

The optimal gene set *G*_optimal_ containing some genes closely related to IBD can be obtained using the mRMR and IFS methods. To further mine for other related genes, we constructed a large network from the PPI data and searched for additional candidate genes in the network.

To construct the network, we downloaded the file “protein.links.v9.1.txt.gz” containing the PPI information from STRING (Search Tool for the Retrieval of Interacting Genes/Proteins, version 9.1, http://www.string-db.org/), from which the human PPI data were extracted by identifying lines starting with “9606.” A total of 2,425,314 human PPIs involving 20,770 proteins represented using Ensembl IDs were obtained. According to STRING (http://string-db.org/) [36, 37], these PPIs are derived from the following sources: (i) genomic context, (ii) high-throughput experiments, (iii) (conserved) coexpression, and (iv) previous knowledge. Thus, the obtained PPIs contained actual PPIs validated using experiments and predicted PPIs, suggesting that they can be used to widely measure the physical and functional relationships between proteins. Each PPI contained two proteins represented using Ensembl IDs and one score that indicates the strength of the interaction with a range between 150 and 999. The constructed network had 20,770 proteins as nodes. Two nodes were adjacent if and only if the corresponding proteins comprise an interaction that is contained in the 2,425,314 human PPIs. Furthermore, the interaction score was also added to the network. Each edge was assigned a weight defined to be 1,000 minus the corresponding interaction score.

### 2.7. SP Approach for Searching for Additional Candidates

Network method is an important type of approaches for investigation of disease genes, such as methods based on guilt-by-association (GBA) [38–40] and Random Walk with Restart (RWR) [41–43]. This section proposed another network method for identifying novel disease genes.

It has been elaborated in some previous studies [44–46] that two proteins in an interaction are more likely to share similar functions. It can be induced that the interactive proteins of the proteins encoded by genes in *G*_optimal_ are also related to IBD. Furthermore, if we consider a series of proteins *p*_1_, *p*_2_,…, *p*_*s*_ such that the consecutive proteins comprise a PPI with a high score and *p*_1_, *p*_*s*_ are proteins encoded by genes in *G*_optimal_, *p*_2_, *p*_3_,…, *p*_*s*−1_ may also be related to IBD. From the construction of the network mentioned in Section 2.6, the corresponding nodes of *p*_1_, *p*_2_,…, *p*_*s*_ may comprise a shortest path connecting *p*_1_ and *p*_*s*_. Therefore, for any two genes in *G*_optimal_, we searched the shortest path connecting these two genes, thereby collecting a number of shortest paths. Because the endpoints of these paths represented proteins encoded by genes in *G*_optimal_, genes on these paths may be related to IBD. Thus, we extracted inner nodes on the obtained shortest paths and their corresponding genes can be obtained. To identify novel genes related to IBD, genes in *G*_optimal_ were excluded from the obtained genes. The remaining genes were called shortest-path genes for convenience. To identify these shortest-path genes, a measurement, namely, the betweenness [47], was recorded for each shortest-path gene, and it was defined to be the number of shortest paths containing the shortest-path gene.

Because some nodes occupied general hubs in the constructed network, the corresponding genes may always be selected even if we searched for the shortest path connecting any pair of randomly selected genes; some of these genes may be selected as the shortest-path genes obtained as described above. In fact, they have few or no associations with IBD. Thus, a permutation test is necessary to control for this type of gene. The procedures used are as follows:

1. Randomly produce 1,000 gene sets, say *G*_1_, *G*_2_,…, *G*_1000_, where the size of each set is the same as that of *G*_optimal_.
2. For each *G*_*i*_, search for all the shortest paths connecting any pair of genes in *G*_*i*_ and count the betweenness of the shortest-path gene based on these paths.
3. A total of 1,000 betweenness scores on 1,000 randomly produced gene sets can be obtained for each shortest-path gene. After comparing the betweenness on *G*_optimal_, we calculate another measurement, the permutation FDR, for each shortest-path gene, which is defined to be “the number of betweenness scores on randomly produced gene sets that was larger than that on *G*_optimal_”/1000.
4. Because it is implied that shortest-path genes with high permutation FDRs are general hubs in the network and not specific to IBD, those with permutation FDRs larger than or equal to 0.05 are excluded. The remaining genes are termed candidate genes.

To select genes with core relationships with IBD from the candidate genes, the human PPIs and their interaction scores were directly used. For each candidate gene* g*, we checked the scores of the interactions between* g *and genes in *G*_optimal_ and selected the maximum value among them as the maximum interaction score of* g*. If a candidate gene has a high maximum interaction score, this suggests that it is highly related to at least one gene in *G*_optimal_, indicating that it is more likely to be related to IBD. As 900 is set to be the threshold of the highest confidence cutoff in STRING, we also set 900 as the threshold for the maximum interaction score; that is, genes with maximum interaction scores no less than 900 were finally selected as the candidate genes in this study.

## 3. Results and Discussion

### 3.1. Results of the mRMR and IFS Methods

The mRMR method was executed on the dataset containing 59 Crohn's disease, 26 ulcerative colitis, and 42 normal samples, and each sample was represented using the expression levels of 12,754 genes, thereby yielding the MaxRel feature list and mRMR feature list, which are provided in Supplementary Material I in Supplementary Material available online at https://doi.org/10.1155/2017/5741948.

**Figure 1:**
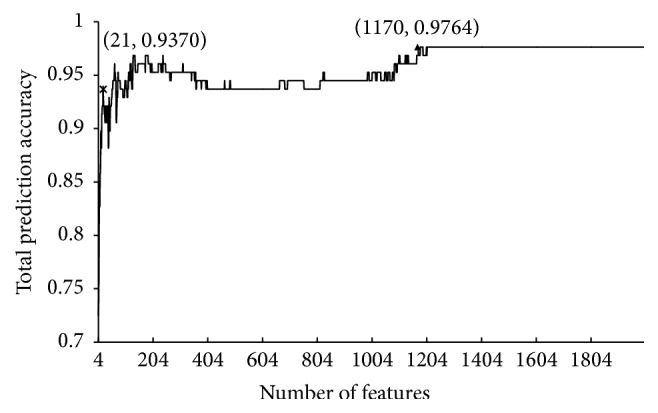
IFS curve. *y*-axis represents the total prediction accuracy, and *x*-axis represents the number of features participating in the classification.

To extract the optimal gene sets for discriminating the samples from two subtypes of IBD and normal samples, the IFS method was used with the mRMR feature list obtained using the mRMR method and SMO as the prediction engine. To reduce computational time and account for the fact that genes with important contributions for discriminating samples from two subtypes of IBD and normal samples are few in number, we only investigated the first 2,000 feature sets. According to the procedures of the IFS method, each feature set can yield four accuracies: three accuracies for three classes and the total prediction accuracy. All of these are provided in Supplementary Material II. Furthermore, an IFS curve was plotted by representing the total prediction accuracy along *y*-axis and the size of the feature set, that is, the number of features participating in the classification, along *x*-axis, as shown in Figure 1. It can be seen that the highest total prediction accuracy was 97.64% using the 1170th feature set. The corresponding accuracies for the three classes were 100%, 92.31%, and 97.62%, respectively. Although the accuracies were quite good, the involved features/genes were too many in number, which is not realistic. By carefully checking the IFS curve shown in Figure 1, we observe a sharp increasing trend with more and more features participating in the classification at the beginning of the curve with a rather high total prediction accuracy (93.70%) using the 21st feature set. Then the curve is unstable; increasing trends and decreasing trend occur in succession. Thus, we believe that the first 21 features in the mRMR feature list are more important for discriminating the samples from two subtypes of IBD and normal samples than others and set the optimal gene set *G*_optimal_ to be the 21st feature set. These 21 genes are listed in Table 1. The associations between these 21 genes and IBD are elaborated in Section 3.4. However, some important IBD-related genes may not be omitted using the mRMR and IFS methods. Based on these genes, the SP approach was applied to discover additional genes related to IBD, which is described in the following sections.

### 3.2. Shortest-Path Genes

**Figure 2:**
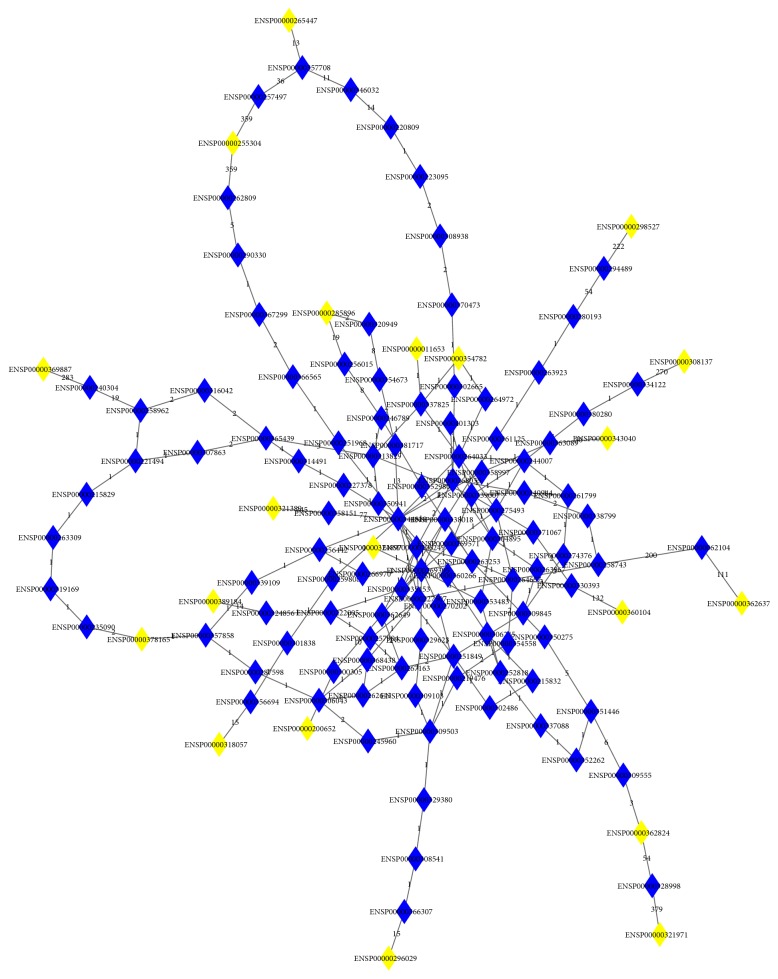
The graph consisting of 190 shortest paths connecting any two genes in the optimal gene set. The yellow diamonds represent genes in the optimal gene set. The blue diamonds represent shortest-path genes. The numbers on the edges represent the edge weights in the network.

As mentioned in Section 3.1, 21 genes were obtained and deemed to be important for discriminating the samples from the two subtypes of IBD and the normal samples. To further identify more candidate genes, we constructed a large network, as described in Section 2.6. These 21 genes were mapped to 20 genes in the network. We searched for all shortest paths connecting any pair of 20 genes, resulting in 190 paths. The graph of these 190 paths is shown in Figure 2, where we can see that there are 110 Ensembl genes on these paths other than the 21 genes obtained in Section 3.1. By mapping to their gene symbols, we obtained 107 shortest-path genes. These genes and their betweenness are listed in Supplementary Material III.

### 3.3. Additional Candidate Genes

According to Section 2.7, a permutation test was executed to exclude general genes in the network. The obtained permutation FDRs of 107 shortest-path genes are also provided in Supplementary Material III. By setting the threshold of the permutation FDR to be 0.05, 57 candidate genes were obtained, which are listed in Supplementary Material IV.

To select the core genes among the 57 candidate genes, the maximum interaction score of each candidate gene was calculated. These values are also provided in Supplementary Material IV. The threshold of the maximum interaction score was set to 900, resulting in 20 candidate genes that are listed in Table 2.

### 3.4. Analysis of Candidate Genes

Based on feature analysis of 59 Crohn's disease, 26 ulcerative colitis, and 42 normal samples, we obtained 21 genes, listed in Table 1, which may be related to IBD and can help distinguish healthy people from those who have two subtypes of IBD. Furthermore, according to the above 21 genes and the SP approach, we obtained additional 20 candidate genes, listed in Table 2. These genes are also thought to be related to IBD. This section provides some evidence for this claim.

We combined two candidate gene sets and analyzed the biological meaning behind them using Functional Annotation Bioinformatics Microarray Analysis (DAVID) (version 6.7, https://david.ncifcrf.gov/) [48]. The obtained results are provided in Supplementary Material V. According to the results yielded by DAVID, crucial gene ontology (GO) terms and KEGG pathways like hsa04660 (T cell receptor signaling pathway), GO: 0001775 (cell activation), and GO: 0045449 (regulation of transcription) were screened out to be enriched by 41 candidate genes. In addition, the results also gave clues for clustering 41 candidate genes into some groups, which provided convenience for analyzing candidate genes.

#### 3.4.1. Candidate Genes Contributing to T Cell Receptor Signaling Pathway (hsa04660)

As mentioned above, IBD is a severe disease induced by inflammation reactions [1]. Considering the core regulatory role of T cells in immune system, it is quite reasonable that various candidate genes contribute to such pathway. Based on SP approach, we identified a specific gene* FOS*. It is also a tumor-associated gene, which encodes a leucine zipper protein that can dimerize with proteins of the JUN family, thereby forming the transcription factor complex AP-1 [49]. Related to crucial pathways such as NF-kB and MAPK, FOS is quite significant in inflammation initiation, especially in the digestive tract [50, 51]. Another calcium-associated gene* PLCG1* was also discovered. PLC1 participates in the intracellular transduction of receptor-mediated tyrosine kinase activators and may participate in the inflammation reaction through a specific function [52, 53].* LCK* (also known as p56lck) is another predicted IBD-related gene that encodes a functional tyrosine kinase. Similar to ZAP70, LCK also regulates the metabolism and maturation of T cells and may further regulate the inflammation process [54, 55]. In terms of IBD, LCK has been reported to be associated with ulcerative colitis but not with Crohn's disease [56]. Our predicted gene* ZAP70* is a protein tyrosine kinase participating in the development and activation of T cells [57, 58]. ZAP70 has been reported to be associated with a specific subtype of IBD, Crohn's disease, but not ulcerative colitis [59]. Therefore, the expression level of ZAP70 can be a useful biomarker for distinguishing different subtypes of IBD.

While based on the mRMR and IFS method, we also identified a group of candidate genes. Among them,* CD247* (rank 3 in the mRMR feature list) and* CD4* (rank 19 in the mRMR feature list) are both crucial genes for T cells and have been confirmed to further regulate the inflammation reaction [60, 61]. Our predicted gene CD4 is characteristically expressed in IBD [62]. However, CD4 has also been reported as a differentially expressed gene in Crohn's disease and ulcerative colitis, and it may further serve as a new biomarker for distinguishing these two diseases [63]. Based on our functional clustering, various screened and predicted genes also are enriched in a similar GO term, GO: 0042101 which describes T cell receptor complex as a cellular component, validating the enrichment of T cell receptor signaling pathway of our screened out IBD associated genes.

#### 3.4.2. Candidate Genes Contributing to Cell Activation (GO: 0001775)

In Table 2, a highly conserved monooxygenase-associated protein* YWHAZ* was identified as a functional protein in intestinal bowel disease. Such gene is a crucial housekeeping gene that has been proven to be a suitable normalizer for bowel inflammation and cancer [60]. As a functional factor of innate immune response, which is also crucial in intestinal tissues,* TLR4* is predicted to be associated with IBD. TLR4 has been reported as a crucial factor in the innate immune barrier of the intestine [61]. Such factors can be activated by specific factors (FFA, etc.) and further induce the initiation of IBD [64, 65]. Another thrombin-associated gene,* F2* (coagulation factor II) was also identified by the SP approach. F2 and THBD are both coagulation-associated genes. The coagulation process is reported to be associated with Crohn's disease but not with ulcerative colitis, which reflects the differences between various subtypes of IBD [66]. There are two major subtypes of IBD: Crohn's disease and ulcerative colitis. Some candidate genes yielded by mRMR and IFS methods may distinguish these two subtypes.* PF4* (rank 5 in the mRMR feature list), a crucial diagnostic biomarker for IBD, has been clearly reported to be overexpressed in Crohn's disease and thought that it does not play a clear role in ulcerative colitis [67, 68]. PF4 can also separate IBD from normal inflammation, which is crucial for diagnosis [69].

#### 3.4.3. Candidate Genes Contributing to Regulation of Transcription (GO: 0045449)

Among the 41 candidate genes, quite a lot of genes contribute to the regulation of transcription, implying the complicated endogenous pathological factors of IBD on multiple levels. Based on mRMR and IFS methods, the candidate gene* ZNF207* (rank 1 in the mRMR feature list), which is a specific microtubule-associated zinc finger protein, may regulate the inflammation of IBD [70]. As a regulator of mitotic chromosome alignment, ZNF207 has been reported to be related to another type of inflammation disorder, chronic obstructive pulmonary disease (COPD). Since both COPD and IBD are localized inflammation involving the mucosal tissue, ZNF207 as our candidate gene may also contribute to inflammatory bowel disease [71]. As a T cell regulator,* EGR3* (rank 8 in the mRMR feature list) was also identified. As a member of the EGR family, EGR3 may be a crucial transcriptional factor for T cells, with high similarity with EGR2 [72].* SLTM* (rank 4 in the mRMR feature list) acts as a general inhibitor of transcription that eventually leads to apoptosis via the regulation of telomere [73]. Because IBD is associated with abnormal cell death, SLTM may participate in IBD through the regulation of the apoptosis of intestinal cells [74].* CNOT8* (rank 14 in the mRMR feature list) is a significant predicted gene that interacts with BTG, the regulator of the cell cycle, especially in B cells [75]. Therefore, CNOT8 may indirectly participate in the intestinal inflammation reaction [75, 76].* TH1L* (rank 13 in the mRMR feature list), as a negative elongation factor complex member C/D (NELFCD), promotes the proliferation of intestinal cells and has been proved to induce carcinoma progression [77]. As a regulator of B cells,* HMGB1* (rank 9 in the mRMR feature list) and its homolog HMGB2 constitute a complex that is differentially expressed in Crohn's disease and ulcerative colitis [78, 79]. Such a complex has also been reported as a new marker of IBD and may be a sensitive marker of mucosal inflammation [80]. As we have mentioned above, our predicted gene CD4 is characteristically expressed in IBD [62]. However, CD4 has also been reported as a differentially expressed gene in Crohn's disease and ulcerative colitis, and it may further serve as a new biomarker for distinguishing these two diseases [63].* UBE2I* (rank 12 in the mRMR feature list) also regulates the proliferation of intestinal cells [81]. Unlike FOLR1, which we will analyze below, UBE2I is a major part of the SUMO ligases and further promotes the proliferation of intestinal cells via multiple means even under pathological conditions [81, 82].

For the candidate genes obtained by the SP approach,* HCFC1* is a functional nuclear activator. As a unique cleavage signal, it has been reported to be associated with cell cycle regulation and may have a specific function in tumorigenesis [83, 84]. As a part of the CCR4-NOT complex,* CNOT1* is a crucial immune associated gene that is a major cellular mRNA deadenylase and has been reported to participate in several processes related to immune reactions [85]. Regulated by the CCR4-NOT complex, a crucial microRNA, miR155, has been reported to be directly associated with inflammation, which may further reveal the tight connection between CNOT1 and the inflammation reaction [86, 87]. Such functional genes may also participate in the initiation of inflammation and tumors.* CNOT4* is also a part of the CCR-NOT complex, and CNOT4 may act similarly to CNOT1 and contribute to the regulation of the immune reaction [85]. As a functional factor of innate immune response, which is also crucial in intestinal tissues,* TRAK1* is a regulatory gene that may be related to endosome-to-lysosome trafficking and EGF-EGFR interaction [88]. Such an EGF-EGFR interaction is definitely associated with the initiation of bowel inflammation [89]. The candidate gene* HDAC1* regulates the acetylation of specific genes and further participates in the regulation of corresponding functions [90]. Gene acetylation and deacetylation are functional regulatory methods for cell metabolism, which have been identified in IBD [91–93]. Therefore, HDAC1 may play a regulatory role in the initiation and progression of intestinal bowel diseases.* BTG1* is a functional regulatory gene associated with cell growth and differentiation. Similar to FASLG, it also regulates the apoptosis of specific target cells and may further regulate specific cytokines associated with inflammation such as IFN-*γ* [94]. Histone deacetylase is commonly used to modify the epigenetic status and regulate gene expression [95].* RUNX1*, known as runt-related transcription factor 1, is quite crucial in the development of normal hematopoiesis as a part of CBF (core binding factor). Associated with T cell function and TGF-*β*, RUNX1 has been proven to be quite crucial in inflammation initiation [96, 97]. Considering the strong relationship between IBD and immune reaction, RUNX1, which regulates the function of T cells, may also participate in the initiation of IBD [98].

#### 3.4.4. Candidate Genes Contributing to Protein Kinase Cascade (GO: 0007243)

Four functional genes have been clustered into such group. Genes like F2, ZAP70, and TLR4 have already been analyzed above. The gene* MARK2* (rank 2 in the mRMR feature list) may also contribute to the initiation and progression of IBD by interfering with the protein kinase cascade. Inflammation is a basic pathological process regulated by the immune system [99]. Therefore, the immune system plays an irreplaceable role in IBD [100]. Several predicted genes have been confirmed to be associated with the immune system and participate in the immune reaction. MARK2 is a serine/threonine-protein kinase that is the major regulator of cell polarity in epithelial cells, including intestinal epithelial cells. Since immune cells in intestinal system have been proven to be regulated by such gene, the abnormal expression and effect of MARK2 may contribute to the unusual activation of focal inflammatory reaction in the digestive system, which may further promote IBD [101].

#### 3.4.5. Candidate Genes Contributing to Intrinsic to Plasma Membrane (GO: 0031226)

Among the candidate genes obtained by the SP approach,* THBD* is an endothelial-specific type I membrane receptor that binds thrombin [102]. As a specific protein in coagulation mechanisms, this receptor has also been reported as a potential inflammation mediator and may have a specific function in IBD [103, 104]. We also predicted a specific member of the TNF family,* FASLG*, as a candidate gene. FASLG has been proven to be involved in the induction of apoptosis triggered by binding to FAS [105]. Members of the TNF family have been widely reported to participate in IBDs by regulating the apoptosis of specific local cells [106, 107].

For candidate genes listed in Table 1,* FOLR1* (rank 6 in the mRMR feature list), the folate receptor, participates in intestinal inflammation via the regulation of folate. Folate is associated with cell apoptosis in bowel tissues and has been reported to be crucial in colonic epithelial cell proliferation implying its potential role in inflammatory bowel diseases [108, 109].* SLC22A4* (rank 18 in the mRMR feature list) is a homolog of SLC22A5, which has been reported to be crucial in Crohn's disease and is also overexpressed in this disease [110]. However, just like SLC22A5, SLC22A4 has not been confirmed to be overexpressed in ulcerative colitis [111, 112]. The genes mentioned above can distinguish IBD subtypes at the genetic level and may serve as new markers for the classification of inflammation in intestinal tissues. As a receptor of significant biological signals,* LEPROT* (rank 10 in the mRMR feature list) encodes a crucial receptor of GH and has been reported to be associated with the initiation of inflammation in the intestine in mice [113]. IBD has been regarded to be the result of immune systematic disorders and autoimmune reactions [1, 100]. Another gene,* CLEC1B* (rank 16 in the mRMR feature list), also participates in the development of IBD via the regulation of the intestinal immune system, especially the proliferation of NK cells and the formation of lymph nodes [114]. Apart from NK cells, activated cell is also a major part of the immune system and has been shown to be related to IBD [115, 116].

#### 3.4.6. Candidate Genes Contributing to Apoptosis (GO: 0006915)

Some candidate genes have been confirmed to participate in the apoptosis processes during the pathological processes of IBD. Apart from genes like SLTM, LCK, F2, FASLG, and BLCAP which we have just analyzed above, the candidate gene* RHOT2* (rank 21 in the mRMR feature list), a mitochondrial GTPase involved in mitochondrial trafficking, has been proven to be crucial regulator of Ca^2+^ in T cells. Thus, RHOT2 may also contribute to IBD [117]. IBD is a common disease involving the digestive system, especially the intestinal tissue [1]. However, IBD has also been shown to be associated with carcinoma in the digestive system, especially colorectal cancer [100]. Several of our predicted genes are also involved in tumor initiation, where cells may have mutated in precancerous lesions, including severe IBD. Most of these genes are related to cell proliferation.* BLCAP* (rank 7 in the mRMR feature list), which was first reported in bladder cancer, regulates the proliferation of cells that are quite common in intestinal tissue of IBD patients [118].

#### 3.4.7. Candidate Genes Contributing to Regulation of Cell Proliferation (GO: 0042127)

Among the candidate genes listed in Table 2, several have been confirmed to contribute to cell proliferation, implying the potential role of that during IBD initiation and progression.* STK11*, a functional serine/threonine kinase, regulates the polarity of cells and may participate in tumor suppression [119]. NF-kB is a crucial transcriptional factor that participates in the inflammation process [120]. STK11 (also known as LKB1) directly regulates the function of NF-kB and is definitely associated with inflammation [121]. STK11 also regulates the proliferation and maturation of intestinal cells, which indirectly reflects the regulatory function of STK11 in intestinal tissues. The calcium binding protein* S100A6* is also on our predicted list, and it is located in the cytoplasm and nucleus of a wide range of cells. S100A6 regulates the progression of the cell cycle and the differentiation of specific cells [122]. Considering the tight relationship between IBD and cancer, some of our predicted genes are also associated with tumor initiation [123]. We also predict as a candidate gene a serine proteinase inhibitor* SERPINE1*, which encodes the principal inhibitor of tissue plasminogen activator (tPA) and urokinase (uPA). Tissue plasminogen activator and urokinase are both associated with inflammation and the process of wound healing [124]. SERPINE1 and proteins in the downstream of its specific pathway have also been reported to be directly associated with IBD as a functional regulator [125, 126]. As a candidate gene, we also predicted an angiogenesis-associated gene* VEGFC*, which regulates angiogenesis and endothelial cell growth [127, 128]. VEGFC has been reported to participate in several intestinal disorders including IBD and some specific digestive tract cancers [129, 130].

#### 3.4.8. Other Candidate Genes

Four candidate genes obtained by mRMR and IFS methods were not clustered into any above group. The candidate gene* ANXA11* (rank 20 in the mRMR feature list) is a predicted gene that regulates the autoimmune reaction. Such a gene has been reported to be related to several autoimmune disorders and may further participate in intestinal inflammation [131]. As a part of the MLL complex,* OGT* (rank 15 in the mRMR feature list) regulates the cell cycle of intestinal cells, including immune cells [132]. Therefore, the abnormity of the OGT gene may induce IBD in various downstream pathways. Another candidate gene,* USPL1* (rank 17 in the mRMR feature list), also participates in intestinal inflammation reaction via the SUMO complex [133]. Large-scale mapping of human protein-protein interactions by mass spectrometry revealed several genes associated with inflammation, especially in the intestine [76]. The last gene,* HIST1H2AC* (rank 11 in the mRMR feature list), is also a candidate gene for cancer. Such gene was first reported in breast cancer and regulates the proliferation of tissue cells, similar to BLCAP [134].

### 3.5. Comparison of Other Methods

To indicate the effectiveness of the proposed method and the reliability of the obtained genes, we compared our method with other methods. Before making the comparison, 77 validated IBD-related genes were retrieved from [135], which are provided in Supplementary Material VI. These genes were used to test the results yielded by our method and other methods.

DisGeNET (Verison 4.0) [136] is a discovery platform that collects gene-disease associations from several public data sources and the literature. Here, it was used to search IBD-related genes. The obtained material is provided in Supplementary Material VII, from which we extracted 100 genes with high confidence (score > 0.1) as the predicted genes of this method. DAVID 6.7 (https://david.ncifcrf.gov/) [48] was employed again to analyze the biological meanings behind the validated genes, predicted genes by our method, and predicted genes by DisGeNET. The enriched gene ontology (GO) terms and KEGG pathways for three gene lists are listed in Supplementary Material V. It can be observed that 209 GO terms and KEGG pathways were enriched by 77 validated genes, while, for predicted genes by our method and DisGeNET, we obtained 154 and 314 GO terms and KEGG pathways, respectively. For the 154 GO terms and KEGG pathways enriched by 41 predicted genes of our method, 51 (51/154 = 33.12%) were also enriched by 77 validated genes, while there were 117 (117/314 = 37.26%) GO terms and KEGG pathways enriched by both 77 validated genes and 100 predicted genes of DisGeNET.

At a first glance, the performance of the DisGeNET is superior to our method. However, our method still has its advantages. According to our method, 21 genes were extracted by analyzing the gene expression profiles using mRMR, IFS, and SMO methods. In fact, these genes can only help us to distinguish two subtypes of IBD (rather than all subtypes of IBD) and normal samples. Thus, they are parts of IBD-related genes even if they are really IBD-related genes. 20 additional candidate genes were further obtained based on these genes, thereby accessing 41 predicted genes. These 41 predicted genes, in fact, are deemed to be related to two subtypes of IBD rather other all IBD subtypes. On the other hand, 100 predicted genes yielded by DisGeNET considered all subtypes of IBD. It is an important reason why DisGeNET gave the better performance. However, the performance of our method is only slightly lower than that of DisGeNET. Therefore, we believe that the proposed method is still quite effective and the obtained genes can be important and reliable materials for the investigation of IBD.

## 4. Conclusions

This contribution provides a novel computational method to identify genes related to IBD, which consists of two main steps: (1) analyzing the gene expression profiles and extracting important genes for IBD and (2) applying the shortest-path approach to the network constructed using protein-protein interactions and identifying additional related genes. By analyzing the obtained genes, it is concluded that they have special relationships with IBD, implying that our method is effective. It is also believed that our method has potential applicability for the investigation of other diseases.

## Supplementary Material

The Supplementary Material consists of seven files. In detail, Supplementary Material I lists the MaxRel feature list and mRMR feature list obtained by mRMR method; Supplementary Material II lists the total prediction accuracy and accuracies for three classes obtained by the IFS method; Supplementary Material III lists 107 shortest path genes and their betweenness and permutation FDRs; Supplementary Material IV lists 57 candidate genes and their maximum interaction scores; Supplementary Material V lists the analysis results of DAVID on candidate genes; Supplementary Material VI lists 77 validated IBD-related genes reported in a paper; Supplementary Material VII lists the results yielded by DisGeNET.

## Figures and Tables

**Table 1:** Twenty-one important genes for IBD obtained using the mRMR and IFS methods.

GO term/KEGG pathway ID	Description	Rank^a^	Gene symbol	Description
hsa04660	T cell receptor signaling pathway	3	CD247	CD247 molecule
		19	CD4	CD4 molecule

GO: 0001775	Cell activation	5	PF4	Platelet factor 4

GO: 0045449	Regulation of transcription	1	ZNF207	Zinc finger protein 207
		8	EGR3	Early growth response 3
		4	SLTM	SAFB-like, transcription modulator
		14	CNOT8	CCR4-NOT transcription complex, subunit 8
		13	TH1L (NELFCD)	Negative elongation factor complex member C/D
		9	HMGB1	High mobility group box 1
		12	UBE2I	Ubiquitin-conjugating enzyme E2I

GO: 0007243	Protein kinase cascade	2	MARK2	MAP/microtubule affinity-regulating kinase 2

GO: 0031226	Intrinsic to plasma membrane	6	FOLR1	Folate receptor 1 (adult)
		18	SLC22A4	Solute carrier family 22 (organic cation/zwitterion transporter), member 4
		10	LEPROT	Leptin receptor overlapping transcript
		16	CLEC1B	C-type lectin domain family 1, member B

GO: 0006915	Apoptosis	21	RHOT2	ras homolog family member T2
		7	BLCAP	Bladder cancer associated protein

Non-grouped genes		20	ANXA11	Annexin A11
		15	OGT	O-linked N-acetylglucosamine (GlcNAc) transferase
		17	USPL1	Ubiquitin specific peptidase like 1
		11	HIST1H2AC	Histone cluster 1, H2ac

a: this column indicates the ranks of related features in the mRMR feature list.

**Table 2:** Twenty candidate genes obtained by SP approach.

GO term/KEGG pathway ID	Description	Gene symbol	Ensembl ID	Description	Betweenness	Permutation FDR	Maximum interaction score	Most related gene in the optimal gene set
hsa04660	T cell receptor signaling pathway	FOS	ENSP00000306245	FBJ murine osteosarcoma viral oncogene homolog	20	0.035	950	CD4
		PLCG1	ENSP00000244007	Phospholipase C, gamma 1	19	0.022	927	CD4
		LCK	ENSP00000337825	LCK protooncogene, Src family tyrosine kinase	21	0.02	999	CD4
		ZAP70	ENSP00000264972	Zeta-chain (TCR) associated protein kinase 70 kDa	16	0.016	999	CD247

GO: 0001775	Cell activation	YWHAZ	ENSP00000309503	Tyrosine 3-monooxygenase/tryptophan 5-monooxygenase activation protein, zeta	19	0.009	962	MARK2
		TLR4	ENSP00000363089	Toll-like receptor 4	19	<0.001	970	HMGB1
		F2	ENSP00000308541	coagulation factor II (thrombin)	19	<0.001	953	PF4

GO: 0045449	Regulation of transcription	HCFC1	ENSP00000309555	Host cell factor C1	36	<0.001	997	OGT
		CNOT1	ENSP00000320949	CCR4-NOT transcription complex, subunit 1	6	0.004	998	CNOT8
		CNOT4	ENSP00000354673	CCR4-NOT transcription complex, subunit 4	6	0.008	987	CNOT8
		TRAK1	ENSP00000328998	Trafficking protein, kinesin binding 1	19	<0.001	946	OGT
		HDAC1	ENSP00000362649	Histone deacetylase 1	19	0.031	967	UBE2I
		BTG1	ENSP00000256015	B-cell translocation gene 1, anti-proliferative	13	0.006	981	CNOT8
		RUNX1	ENSP00000300305	Runt-related transcription factor 1	19	<0.001	927	SLC22A4

GO: 0031226	Intrinsic to plasma membrane	THBD	ENSP00000366307	Thrombomodulin	19	<0.001	985	PF4
		FASLG	ENSP00000356694	Fas ligand (TNF superfamily, member 6)	19	<0.001	985	EGR3

GO: 0042127	Regulation of cell proliferation	STK11	ENSP00000324856	Serine/threonine kinase 11	19	<0.001	986	MARK2
		S100A6	ENSP00000357708	S100 calcium binding protein A6	19	<0.001	987	ANXA11
		SERPINE1	ENSP00000223095	Serpin peptidase inhibitor, clade E (nexin, plasminogen activator inhibitor type 1), member 1	18	0.034	933	PF4
		VEGFC	ENSP00000280193	Vascular endothelial growth factor C	19	<0.001	919	PF4

## References

[1] Mulder D. J., Noble A. J., Justinich C. J., Duffin J. M. (2014). A tale of two diseases: the history of inflammatory bowel disease. *Journal of Crohn's and Colitis*.

[2] Barbara G., Cremon C., Stanghellini V. (2014). Inflammatory bowel disease and irritable bowel syndrome: similarities and differences. *Current Opinion in Gastroenterology*.

[3] Bassotti G., Antonelli E., Villanacci V., Salemme M., Coppola M., Annese V. (2014). Gastrointestinal motility disorders in inflammatory bowel diseases. *World Journal of Gastroenterology*.

[4] Zippi M., Corrado C., Pica R. (2014). Extraintestinal manifestations in a large series of Italian inflammatory bowel disease patients. *World Journal of Gastroenterology*.

[5] Marineata A., Rezus E., Mihai C., Prelipcean C. C. (2014). Extra intestinal manifestations and complications in inflammatory bowel disease. *Revista Medico-Chirurgicala a Societatii de Medici si Naturalisti din Iasi*.

[6] Lakatos P. L., Lakatos L., Kiss L. S., Peyrin-Biroulet L., Schoepfer A., Vavricka S. (2012). Treatment of extraintestinal manifestations in inflammatory bowel disease. *Digestion*.

[7] Singh S., Kullo I. J., Pardi D. S., Loftus E. V. (2015). Epidemiology, risk factors and management of cardiovascular diseases in IBD. *Nature Reviews Gastroenterology & Hepatology*.

[8] Ruel J., Ruane D., Mehandru S., Gower-Rousseau C., Colombel J.-F. (2014). IBD across the age spectrum—is it the same disease?. *Nature Reviews Gastroenterology and Hepatology*.

[9] Jaźwińska-Tarnawska E., Jęśkowiak I., Waszczuk E. (2015). Genetic polymorphism of ABCB1 gene (C3435T) in patients with inflammatory bowel diseases. Is there any gender dependency?. *Pharmacological Reports*.

[10] Jakobsen C., Cleynen I., Andersen P. S. (2014). Genetic susceptibility and genotype-phenotype association in 588 Danish children with inflammatory bowel disease. *Journal of Crohn's & Colitis*.

[11] Nguyen G. C., Chong C. A., Chong R. Y. (2014). National estimates of the burden of inflammatory bowel disease among racial and ethnic groups in the United States. *Journal of Crohn's and Colitis*.

[12] Zhang Y., Li Y. Y. (2014). Inflammatory bowel disease: pathogenesis. *World Journal of Gastroenterology*.

[13] Flanagan P., Campbell B. J., Rhodes J. M. (2011). Bacteria in the pathogenesis of inflammatory bowel disease. *Biochemical Society Transactions*.

[14] Lees C. W., Barrett J. C., Parkes M., Satsangi J. (2011). New IBD genetics: common pathways with other diseases. *Gut*.

[15] Glas J., Seiderer J., Wagner J. (2012). Analysis of IL12B gene variants in inflammatory bowel disease. *PLOS ONE*.

[16] John G., Hegarty J. P., Yu W. (2011). NKX2-3 variant rs11190140 is associated with IBD and alters binding of NFAT. *Molecular Genetics and Metabolism*.

[17] VanDussen K. L., Liu T. C., Li D. (2014). Genetic variants synthesize to produce paneth cell phenotypes that define subtypes of Crohn's disease. *Gastroenterology*.

[18] Kabashima K., Saji T., Murata T. (2002). The prostaglandin receptor EP4 suppresses colitis, mucosal damage and CD4 cell activation in the gut. *Journal of Clinical Investigation*.

[19] Patman G. (2014). Crohn's disease: suppression of p21Rac1 signalling contributes to skip-lesion phenotype in Crohn's disease. *Nature Reviews Gastroenterology & Hepatology*.

[20] Rumessen J. J. (1996). Ultrastructure of interstitial cells of Cajal at the colonic submuscular border in patients with ulcerative colitis. *Gastroenterology*.

[21] Hampe J., Franke A., Rosenstiel P. (2007). A genome-wide association scan of nonsynonymous SNPs identifies a susceptibility variant for Crohn disease in ATG16L1. *Nature Genetics*.

[22] Parkes M., Barrett J. C., Prescott N. J. (2007). Sequence variants in the autophagy gene IRGM and multiple other replicating loci contribute to Crohn's disease susceptibility. *Nature Genetics*.

[23] Hisamatsu T., Suzuki M., Reinecker H.-C., Nadeau W. J., McCormick B. A., Podolsky D. K. (2003). CARD15/NOD2 functions as an antibacterial factor in human intestinal epithelial cells. *Gastroenterology*.

[24] Leung E., Hong J., Fraser A. G., Merriman T. R., Vishnu P., Krissansen G. W. (2006). Polymorphisms in the organic cation transporter genes SLC22A4 and SLC22A5 and Crohn's disease in a New Zealand Caucasian cohort. *Immunology and Cell Biology*.

[25] McCole D. F. (2012). Regulation of epithelial barrier function by the inflammatory bowel disease candidate gene, PTPN2. *Annals of the New York Academy of Sciences*.

[26] Peng H., Long F., Ding C. (2005). Feature selection based on mutual information: criteria of max-dependency, max-relevance, and min-redundancy. *IEEE Transactions on Pattern Analysis and Machine Intelligence*.

[27] Platt J. (1998). *Fast Training of Support Vector Machines Using Sequential Minimal Optimization*.

[28] Keerthi S. S., Shevade S. K., Bhattacharyya C., Murthy K. R. K. (2001). Improvements to Platt's SMO algorithm for SVM classifier design. *Neural Computation*.

[29] Burczynski M. E., Peterson R. L., Twine N. C. (2006). Molecular classification of Crohn's disease and ulcerative colitis patients using transcriptional profiles in peripheral blood mononuclear cells. *The Journal of Molecular Diagnostics*.

[30] Chen L., Chu C., Feng K. (2016). Predicting the types of metabolic pathway of compounds using molecular fragments and sequential minimal optimization. *Combinatorial Chemistry & High Throughput Screening*.

[31] Mohabatkar H., Mohammad Beigi M., Esmaeili A. (2011). Prediction of GABAA receptor proteins using the concept of Chou's pseudo-amino acid composition and support vector machine. *Journal of Theoretical Biology*.

[32] Witten I. H., Frank E. (2005). *Data Mining: Practical Machine Learning Tools and Techniques*.

[33] Kohavi R. A study of cross-validation and bootstrap for accuracy estimation and model selection.

[34] Chen L., Lu J., Zhang N., Huang T., Cai Y.-D. (2014). A hybrid method for prediction and repositioning of drug Anatomical Therapeutic Chemical classes. *Molecular BioSystems*.

[35] Chen L., Zeng W.-M., Cai Y.-D., Feng K.-Y., Chou K.-C. (2012). Predicting anatomical therapeutic chemical (ATC) classification of drugs by integrating chemical-chemical interactions and similarities. *PLOS ONE*.

[36] von Mering C., Huynen M., Jaeggi D., Schmidt S., Bork P., Snel B. (2003). STRING: a database of predicted functional associations between proteins. *Nucleic Acids Research*.

[37] Franceschini A., Szklarczyk D., Frankild S. (2013). STRING v9.1: protein-protein interaction networks, with increased coverage and integration. *Nucleic Acids Research*.

[38] Oti M., Snel B., Huynen M. A., Brunner H. G. (2006). Predicting disease genes using protein-protein interactions. *Journal of Medical Genetics*.

[39] Krauthammer M., Kaufmann C. A., Gilliam T. C., Rzhetsky A. (2004). Molecular triangulation: bridging linkage and molecular-network information for identifying candidate genes in Alzheimer's desease. *Proceedings of the National Academy of Sciences of the United States of America*.

[40] Franke L., van Bakel H., Fokkens L., de Jong E. D., Egmont-Petersen M., Wijmenga C. (2006). Reconstruction of a functional human gene network, with an application for prioritizing positional candidate genes. *The American Journal of Human Genetics*.

[41] Köhler S., Bauer S., Horn D., Robinson P. N. (2008). Walking the Interactome for Prioritization of Candidate Disease Genes. *The American Journal of Human Genetics*.

[42] Jiang R., Gan M., He P. (2011). Constructing a gene semantic similarity network for the inference of disease genes. *BMC Systems Biology*.

[43] Shi H., Xu J., Zhang G. (2013). Walking the interactome to identify human miRNA-disease associations through the functional link between miRNA targets and disease genes. *BMC Systems Biology*.

[44] Jiang M., Chen Y., Zhang Y. (2013). Identification of hepatocellular carcinoma related genes with k-th shortest paths in a protein–protein interaction network. *Molecular BioSystems*.

[45] Chen L., Xing Z. H., Huang T., Shu Y., Huang G., Li H.-P. (2016). Application of the shortest path algorithm for the discovery of breast cancer-related genes. *Current Bioinformatics*.

[46] Gui T., Dong X., Li R., Li Y., Wang Z. (2015). Identification of hepatocellular carcinoma-related genes with a machine learning and network analysis. *Journal of Computational Biology*.

[47] Kitsak M., Havlin S., Paul G., Riccaboni M., Pammolli F., Stanley H. E. (2007). Betweenness centrality of fractal and nonfractal scale-free model networks and tests on real networks. *Physical Review E—Statistical, Nonlinear, and Soft Matter Physics*.

[48] Huang D. W., Sherman B. T., Lempicki R. A. (2009). Systematic and integrative analysis of large gene lists using DAVID bioinformatics resources. *Nature Protocols*.

[49] Li C., Li H., Wang S. (2015). The c-Fos and c-Jun from *Litopenaeus vannamei* play opposite roles in *Vibrio parahaemolyticus* and white spot syndrome virus infection. *Developmental and Comparative Immunology*.

[50] Thummuri D., Jeengar M. K., Shrivastava S. (2015). Thymoquinone prevents RANKL-induced osteoclastogenesis activation and osteolysis in an in vivo model of inflammation by suppressing NF-KB and MAPK Signalling. *Pharmacological Research*.

[51] Welch M. G., Margolis K. G., Li Z., Gershon M. D. (2014). Oxytocin regulates gastrointestinal motility, inflammation, macromolecular permeability, and mucosal maintenance in mice. *American Journal of Physiology—Gastrointestinal and Liver Physiology*.

[52] Auesukaree C., Tochio H., Shirakawa M., Kaneko Y., Harashima S. (2005). Plc1p, Arg82p, and Kcs1p, enzymes involved in inositol pyrophosphate synthesis, are essential for phosphate regulation and polyphosphate accumulation in Saccharomyces cerevisiae. *Journal of Biological Chemistry*.

[53] Engelberg D., Perlman R., Levitzki A. (2014). Transmembrane signaling in *Saccharomyces cerevisiae* as a model for signaling in metazoans: state of the art after 25 years. *Cellular Signalling*.

[54] Chiang Y. J., Hodes R. J. (2015). Regulation of T cell development by c-Cbl: essential role of Lck. *International Immunology*.

[55] Perron M. D., Chowdhury S., Aubry I., Purisima E., Tremblay M. L., Saragovi H. U. (2014). Allosteric noncompetitive small molecule selective inhibitors of CD45 tyrosine phosphatase suppress T-cell receptor signals and inflammation in vivo. *Molecular Pharmacology*.

[56] Toy L. S., Yio X. Y., Lin A., Honig S., Mayer L. (1997). Defective expression of gp180, a novel CD8 ligand on intestinal epithelial cells, in inflammatory bowel disease. *Journal of Clinical Investigation*.

[57] Liao Z., Zhou L., Wang C. (2015). Characteristics of TCR*ζ*, ZAP-70, and FcɛRI*γ* Gene Expression in Patients with T- and NK/T-Cell Lymphoma. *DNA and Cell Biology*.

[58] Sinclair C., Ono M., Seddon B. (2015). A Zap70-dependent feedback circuit is essential for efficient selection of CD4 lineage thymocytes. *Immunology and Cell Biology*.

[59] Bouzid D., Fourati H., Amouri A. (2013). Association of ZAP70 and PTPN6, but not BANK1 or CLEC2D, with inflammatory bowel disease in the tunisian population. *Genetic Testing and Molecular Biomarkers*.

[60] Krzystek-Korpacka M., Diakowska D., Bania J., Gamian A. (2014). Expression stability of common housekeeping genes is differently affected by bowel inflammation and cancer: implications for finding suitable normalizers for inflammatory bowel disease studies. *Inflammatory Bowel Diseases*.

[61] Wang W., Xia T., Yu X. (2015). Wogonin suppresses inflammatory response and maintains intestinal barrier function via TLR4-MyD88-TAK1-mediated NF-*κ*B pathway in vitro. *Inflammation Research*.

[62] De Almeida C. S., Andrade-Oliveira V., Câmara N. O. S., Jacysyn J. F., Faquim-Mauro E. L. (2015). Crotoxin from Crotalus durissus terrificus is able to down-modulate the acute intestinal inflammation in mice. *PLoS ONE*.

[63] Brandhorst G., Weigand S., Eberle C. (2013). CD4^+^ immune response as a potential biomarker of patient reported inflammatory bowel disease (IBD) activity. *Clinica Chimica Acta*.

[64] Gupta R. A., Motiwala M. N., Dumore N. G., Danao K. R., Ganjare A. B. (2015). Effect of piperine on inhibition of FFA induced TLR4 mediated inflammation and amelioration of acetic acid induced ulcerative colitis in mice. *Journal of Ethnopharmacology*.

[65] Cao A. T., Yao S., Stefka A. T. (2014). TLR4 regulates IFN-*γ* and IL-17 production by both thymic and induced Foxp3+ Tregs during intestinal inflammation. *Journal of Leukocyte Biology*.

[66] Kohoutova D., Pecka M., Cihak M., Cyrany J., Maly J., Bures J. (2014). Prevalence of hypercoagulable disorders in inflammatory bowel disease. *Scandinavian Journal of Gastroenterology*.

[67] Bennike T., Birkelund S., Stensballe A., Andersen V. (2014). Biomarkers in inflammatory bowel diseases: current status and proteomics identification strategies. *World Journal of Gastroenterology*.

[68] Meuwis M.-A., Fillet M., Lutteri L. (2008). Proteomics for prediction and characterization of response to infliximab in Crohn's disease: a pilot study. *Clinical Biochemistry*.

[69] Meuwis M.-A., Fillet M., Geurts P. (2007). Biomarker discovery for inflammatory bowel disease, using proteomic serum profiling. *Biochemical Pharmacology*.

[70] Jiang H., He X., Wang S. (2014). A microtubule-associated zinc finger protein, BuGZ, regulates mitotic chromosome alignment by ensuring Bub3 stability and kinetochore targeting. *Developmental Cell*.

[71] Bhattacharya S., Srisuma S., DeMeo D. L. (2009). Molecular biomarkers for quantitative and discrete COPD phenotypes. *American Journal of Respiratory Cell and Molecular Biology*.

[72] Okamura T., Fujio K., Shibuya M. (2009). CD4^+^CD25^−^LAG3^+^ regulatory T cells controlled by the transcription factor Egr-2. *Proceedings of the National Academy of Sciences of the United States of America*.

[73] Giannone R. J., McDonald H. W., Hurst G. B., Shen R.-F., Wang Y., Liu Y. (2010). The protein network surrounding the human telomere repeat binding factors TRF1, TRF2, and POT1. *PLoS ONE*.

[74] Zemljic M., Pejkovic B., Krajnc I., Lipovsek S. (2014). Biological pathways involved in the development of infammatory bowel disease. *Wiener Klinische Wochenschrift*.

[75] Du Y., Liu P., Zang W. (2015). BTG3 upregulation induces cell apoptosis and suppresses invasion in esophageal adenocarcinoma. *Molecular and Cellular Biochemistry*.

[76] Ewing R. M., Chu P., Elisma F. (2007). Large-scale mapping of human protein–protein interactions by mass spectrometry. *Molecular Systems Biology*.

[77] Carvalho B., Postma C., Mongera S. (2009). Multiple putative oncogenes at the chromosome 20q amplicon contribute to colorectal adenoma to carcinoma progression. *Gut*.

[78] McDonnell M., Liang Y., Noronha A. (2011). Systemic toll-like receptor ligands modify B-cell responses in human inflammatory bowel disease. *Inflammatory Bowel Diseases*.

[79] Vitali R., Stronati L., Negroni A. (2011). Fecal HMGB1 is a novel marker of intestinal mucosal inflammation in pediatric inflammatory bowel disease. *The American Journal of Gastroenterology*.

[80] Takaishi H., Kanai T., Nakazawa A. (2012). Anti-high mobility group box 1 and box 2 non-histone chromosomal proteins (HMGB1/HMGB2) antibodies and anti-Saccharomyces cerevisiae antibodies (ASCA): accuracy in differentially diagnosing UC and CD and correlation with inflammatory bowel disease phenotype. *Journal of Gastroenterology*.

[81] Demarque M. D., Nacerddine K., Neyretkahn H. (2011). Sumoylation by Ubc9 regulates the stem cell compartment and structure and function of the intestinal epithelium in mice. *Gastroenterology*.

[82] Belaguli N. S., Zhang M., Garcia A.-H., Berger D. H. (2012). PIAS1 Is a GATA4 SUMO ligase that regulates GATA4-dependent intestinal promoters independent of SUMO ligase activity and GATA4 sumoylation. *PLoS ONE*.

[83] Zhou P., Wang Z., Yuan X. (2013). Mixed Lineage Leukemia 5 (MLL5) protein regulates cell cycle progression and E2F1-responsive gene expression via association with Host Cell Factor-1 (HCF-1). *The Journal of Biological Chemistry*.

[84] Machida Y. J., Machida Y., Vashisht A. A., Wohlschlegel J. A., Dutta A. (2009). The deubiquitinating enzyme BAP1 regulates cell growth via interaction with HCF-1. *The Journal of Biological Chemistry*.

[85] Chapat C., Corbo L. (2014). Novel roles of the CCR4-NOT complex. *Wiley Interdisciplinary Reviews: RNA*.

[86] Prabowo A. S., van Scheppingen J., Iyer A. M. (2015). Differential expression and clinical significance of three inflammation-related microRNAs in gangliogliomas. *Journal of Neuroinflammation*.

[87] Elton T. S., Selemon H., Elton S. M., Parinandi N. L. (2013). Regulation of the MIR155 host gene in physiological and pathological processes. *Gene*.

[88] Loss O., Stephenson F. A. (2015). Localization of the kinesin adaptor proteins trafficking kinesin proteins 1 and 2 in primary cultures of hippocampal pyramidal and cortical neurons. *Journal of Neuroscience Research*.

[89] Isidro R. A., Cruz M. L., Isidro A. A. (2015). Immunohistochemical expression of SP-NK-1R-EGFR pathway and VDR in colonic inflammation and neoplasia. *World Journal of Gastroenterology*.

[90] Chen P.-J., Huang C., Meng X.-M., Li J. (2015). Epigenetic modifications by histone deacetylases: biological implications and therapeutic potential in liver fibrosis. *Biochimie*.

[91] Felice C., Lewis A., Armuzzi A., Lindsay J. O., Silver A. (2015). Review article: selective histone deacetylase isoforms as potential therapeutic targets in inflammatory bowel diseases. *Alimentary Pharmacology and Therapeutics*.

[92] Lee I. A., Kamba A., Low D., Mizoguchi E. (2014). Novel methylxanthine derivative-mediated anti-inflammatory effects in inflammatory bowel disease. *World Journal of Gastroenterology*.

[93] Garcia-Maurino S., Alcaide A., Dominguez C. (2012). Pharmacological control of autophagy: therapeutic perspectives in inflammatory bowel disease and colorectal cancer. *Current Pharmaceutical Design*.

[94] Lee H., Cha S., Lee M.-S., Cho G. J., Choi W. S., Suk K. (2003). Role of antiproliferative B cell translocation gene-1 as an apoptotic sensitizer in activation-induced cell death of brain microglia. *Journal of Immunology*.

[95] Hamm C. A., Costa F. F. (2015). Epigenomes as therapeutic targets. *Pharmacology & Therapeutics*.

[96] Liu H., Cao A. T., Feng T. (2015). TGF-*β* converts Th1 cells into Th17 cells through stimulation of Runx1 expression. *European Journal of Immunology*.

[97] Wong W. F., Kohu K., Nakamura A. (2012). Runx1 deficiency in CD4^+^ T cells causes fatal autoimmune inflammatory lung disease due to spontaneous hyperactivation of cells. *Journal of Immunology*.

[98] Christophi G. P., Rong R., Holtzapple P. G., Massa P. T., Landas S. K. (2012). Immune markers and differential signaling networks in ulcerative colitis and Crohn's disease. *Inflammatory Bowel Diseases*.

[99] Salisbury D., Bronas U. (2014). Inflammation and immune system contribution to the etiology of atherosclerosis: mechanisms and methods of assessment. *Nursing Research*.

[100] Dunkin D., Mehandru S., Colombel J.-F. (2014). Immune cell therapy in IBD. *Digestive Diseases*.

[101] Hurov J. B., Stappenbeck T. S., Zmasek C. M. (2001). Immune system dysfunction and autoimmune disease in mice lacking Emk (Par-1) protein kinase. *Molecular and Cellular Biology*.

[102] Miwa Y., Yazaki S., Iwamoto M. (2015). Functional difference between membrane-bound and soluble human thrombomodulin. *Transplantation*.

[103] Soult M. C., Dobrydneva Y., Wahab K. H., Britt L. D., Sullivan C. J. (2014). Outer membrane vesicles alter inflammation and coagulation mediators. *Journal of Surgical Research*.

[104] Pekow J., Dougherty U., Huang Y. (2013). Gene signature distinguishes patients with chronic ulcerative colitis harboring remote neoplastic lesions. *Inflammatory Bowel Diseases*.

[105] Lettau M., Paulsen M., Kabelitz D., Janssen O. (2009). FasL expression and reverse signalling. *Results and Problems in Cell Differentiation*.

[106] Ślebioda T. J., Kmieć Z. (2014). Tumour necrosis factor superfamily members in the pathogenesis of inflammatory bowel disease. *Mediators of Inflammation*.

[107] Ben Aleya W., Sfar I., Mouelhi L. (2009). Association of Fas/Apo1 gene promoter (-670 A/G) polymorphism in Tunisian patients with IBD. *World Journal of Gastroenterology*.

[108] Antunes C. V., Hallack Neto A. E., Nascimento C. R. (2015). Anemia in Inflammatory bowel disease outpatients: prevalence, risk factors, and etiology. *BioMed Research International*.

[109] Crott J. W., Liu Z., Keyes M. K. (2008). Moderate folate depletion modulates the expression of selected genes involved in cell cycle, intracellular signaling and folate uptake in human colonic epithelial cell lines. *Journal of Nutritional Biochemistry*.

[110] Pochini L., Scalise M., Galluccio M., Pani G., Siminovitch K. A., Indiveri C. (2012). The human OCTN1 (SLC22A4) reconstituted in liposomes catalyzes acetylcholine transport which is defective in the mutant L503F associated to the Crohn's disease. *Biochimica et Biophysica Acta—Biomembranes*.

[111] Repnik K., Potočnik U. (2011). Haplotype in the IBD5 region is associated with refractory Crohn's disease in Slovenian patients and modulates expression of the SLC22A5 gene. *Journal of Gastroenterology*.

[112] Sarlos P., Varszegi D., Csongei V. (2014). Susceptibility to ulcerative colitis in Hungarian patients determined by gene-gene interactions. *World Journal of Gastroenterology*.

[113] Gove M. E., Rhodes D. H., Pini M. (2008). Role of leptin receptor-induced STAT3 signaling in modulation of intestinal and hepatic inflammation in mice. *Journal of Leukocyte Biology*.

[114] Benezech C., Nayar S., Finney B. A. (2014). CLEC-2 is required for development and maintenance of lymph nodes. *Blood*.

[115] Shin J., Yoon I., Lim J. (2015). CD4+VEGFR1HIGH T cell as a novel Treg subset regulates inflammatory bowel disease in lymphopenic mice. *Cellular and Molecular Immunology*.

[116] Maeda S., Ohno K., Fujiwara-Igarashi A., Uchida K., Tsujimoto H. (2016). Changes in Foxp3-positive regulatory T cell number in the intestine of dogs with idiopathic inflammatory bowel disease and intestinal lymphoma. *Veterinary Pathology*.

[117] Di Sabatino A., Rovedatti L., Kaur R. (2009). Targeting gut T cell Ca2+ release-activated Ca2+ channels inhibits T cell cytokine production and T-box transcription factor T-bet in inflammatory bowel disease. *Journal of Immunology*.

[118] Gromova I., Gromov P., Kroman N. (2012). Immunoexpression analysis and prognostic value of BLCAP in breast cancer. *PLoS ONE*.

[119] Lo A. K.-F., Lo K.-W., Ko C.-W., Young L. S., Dawson C. W. (2013). Inhibition of the LKB1-AMPK pathway by the Epstein-Barr virus-encoded LMP1 promotes proliferation and transformation of human nasopharyngeal epithelial cells. *The Journal of Pathology*.

[120] Verstrepen L., Beyaert R. (2014). Receptor proximal kinases in NF-*κ*B signaling as potential therapeutic targets in cancer and inflammation. *Biochemical Pharmacology*.

[121] Liu Z., Zhang W., Zhang M., Zhu H., Moriasi C., Zou M. (2015). Liver kinase B1 suppresses lipopolysaccharide-induced nuclear factor *κ*B (NF-*κ*B) activation in macrophages. *Journal of Biological Chemistry*.

[122] Calvo F. Q., Fillet M., De Seny D. (2009). Biomarker discovery in asthma-related inflammation and remodeling. *Proteomics*.

[123] Beaugerie L. (2014). IBD and increased risk of cancer: what is the reality?. *La Revue de l'Infirmière*.

[124] Montesinos M. C., Desai-Merchant A., Cronstein B. N. (2015). Promotion of wound healing by an agonist of adenosine A2A receptor is dependent on tissue plasminogen activator. *Inflammation*.

[125] Shaghaghi Z., Bonyadi M., Somi M. H., Khoshbaten M. (2014). Association of plasminogen activator inhibitor-1 gene polymorphism with inflammatory bowel disease in Iranian Azeri Turkish patients. *Saudi Journal of Gastroenterology*.

[126] Koutroubakis I. E., Sfiridaki A., Tsiolakidou G., Coucoutsi C., Theodoropoulou A., Kouroumalis E. A. (2008). Plasma thrombin-activatable fibrinolysis inhibitor and plasminogen activator inhibitor-1 levels in inflammatory bowel disease. *European Journal of Gastroenterology and Hepatology*.

[127] Balboa-Beltran E., Fernández-Seara M. J., Pérez-Muñuzuri A. (2014). A novel stop mutation in the vascular endothelial growth factor-C gene (VEGFC) results in Milroy-like disease. *Journal of Medical Genetics*.

[128] Le Guen L., Karpanen T., Schulte D. (2014). Ccbe1 regulates Vegfc-mediated induction of Vegfr3 signaling during embryonic lymphangiogenesis. *Development*.

[129] Tacconi C., Correale C., Gandelli A. (2015). Vascular endothelial growth factor C disrupts the endothelial lymphatic barrier to promote colorectal cancer invasion. *Gastroenterology*.

[130] D’Alessio S., Correale C., Tacconi C. (2014). VEGF-C-dependent stimulation of lymphatic function ameliorates experimental inflammatory bowel disease. *Journal of Clinical Investigation*.

[131] Jorgensen C. S., Levantino G., Houen G. (2000). Determination of autoantibodies to annexin XI in systemic autoimmune diseases. *Lupus*.

[132] Heuser M., Yap D. B., Leung M. (2009). Loss of MII5 results in pleiotropic hematopoietic defects, reduced neutrophil immune function, and extreme sensitivity to DNA demethylation. *Blood*.

[133] Schulz S., Chachami G., Kozaczkiewicz L. (2012). Ubiquitin-specific protease-like 1 (USPL1) is a SUMO isopeptidase with essential, non-catalytic functions. *EMBO Reports*.

[134] Pärssinen J., Alarmo E.-L., Khan S., Karhu R., Vihinen M., Kallioniemi A. (2008). Identification of differentially expressed genes after PPM1D silencing in breast cancer. *Cancer Letters*.

[135] Liu J. Z., van Sommeren S., Huang H. (2015). Association analyses identify 38 susceptibility loci for inflammatory bowel disease and highlight shared genetic risk across populations. *Nature Genetics*.

[136] Piñero J., Queralt-Rosinach N., Bravo À. (2015). DisGeNET: a discovery platform for the dynamical exploration of human diseases and their genes. *Database*.

