# Supplementary material for: Identification of Candidate Genes Related to Inflammatory Bowel Disease Using Minimum Redundancy Maximum Relevance, Incremental Feature Selection, and the Shortest-Path Approach
Source: Biomed Res Int. 2017 Feb 14;2017:5741948. doi: 10.1155/2017/5741948 (PMC5331171; doi:10.1155/2017/5741948)
Supplement: Supplementary file 1 — The Supplementary Material consists of seven files. In detail, Supplementary Material I lists the MaxRel feature list and mRMR feature list obtained by mRMR method; Supplementary Material II lists the total prediction accuracy and accuracies for three classes obtained by the IFS method; Supplementary Material III lists 107 shortest path genes and their betweenness and permutation FDRs; Supplementary Material IV lists 57 candidate genes and their maximum interaction scores; Supplementary Material V lists the analysis results of DAVID on candidate genes; Supplementary Material VI lists 77 validated IBD-related genes reported in a paper; Supplementary Material VII lists the results yielded by DisGeNET. [file 5741948.f1.zip › Supp-III.docx]

**Supplementary Material III.** 107 shortest path genes and their betweenness and permutation FDRs

| **Gene symbol** | **Ensembl ID** | **Betweenness** | **Permutation FDR** |
| --- | --- | --- | --- |
| NNAT | ENSP00000062104 | 19 | <0.001 |
| LUC7L3 | ENSP00000240304 | 19 | <0.001 |
| VEGFC | ENSP00000280193 | 19 | <0.001 |
| PDPN | ENSP00000294489 | 19 | <0.001 |
| RUNX1 | ENSP00000300305 | 19 | <0.001 |
| FADD | ENSP00000301838 | 19 | <0.001 |
| F2 | ENSP00000308541 | 19 | <0.001 |
| HCFC1 | ENSP00000309555 | 36 | <0.001 |
| STK11 | ENSP00000324856 | 19 | <0.001 |
| TRAK1 | ENSP00000328998 | 19 | <0.001 |
| LEPR | ENSP00000330393 | 19 | <0.001 |
| FGF3 | ENSP00000334122 | 19 | <0.001 |
| WDR5 | ENSP00000351446 | 36 | <0.001 |
| FASLG | ENSP00000356694 | 19 | <0.001 |
| S100A6 | ENSP00000357708 | 19 | <0.001 |
| HIST2H2BE | ENSP00000358151 | 19 | <0.001 |
| TLR4 | ENSP00000363089 | 19 | <0.001 |
| THBD | ENSP00000366307 | 19 | <0.001 |
| SRSF1 | ENSP00000258962 | 19 | 0.001 |
| KDR | ENSP00000263923 | 19 | 0.001 |
| IL6 | ENSP00000258743 | 19 | 0.002 |
| ELL | ENSP00000262809 | 18 | 0.002 |
| HNRNPA0 | ENSP00000316042 | 17 | 0.002 |
| IRAK1 | ENSP00000358997 | 19 | 0.002 |
| CDC37 | ENSP00000222005 | 19 | 0.003 |
| RIPK1 | ENSP00000259808 | 19 | 0.003 |
| GP1BA | ENSP00000329380 | 19 | 0.003 |
| FGFR1 | ENSP00000380280 | 19 | 0.003 |
| CNOT1 | ENSP00000320949 | 6 | 0.004 |
| JUND | ENSP00000252818 | 28 | 0.005 |
| MEN1 | ENSP00000337088 | 28 | 0.005 |
| BTG1 | ENSP00000256015 | 13 | 0.006 |
| SNF8 | ENSP00000290330 | 18 | 0.006 |
| ANXA1 | ENSP00000257497 | 1 | 0.007 |
| CNOT4 | ENSP00000354673 | 6 | 0.008 |
| VEGFA | ENSP00000361125 | 19 | 0.008 |
| YWHAZ | ENSP00000309503 | 19 | 0.009 |
| ANXA2 | ENSP00000346032 | 18 | 0.01 |
| UBE2D2 | ENSP00000381717 | 6 | 0.011 |
| VPS36 | ENSP00000367299 | 18 | 0.015 |
| ZAP70 | ENSP00000264972 | 16 | 0.016 |
| PLAT | ENSP00000220809 | 18 | 0.017 |
| VPS28 | ENSP00000366565 | 18 | 0.019 |
| LCK | ENSP00000337825 | 21 | 0.02 |
| PLCG1 | ENSP00000244007 | 19 | 0.022 |
| IL6ST | ENSP00000338799 | 2 | 0.029 |
| HDAC1 | ENSP00000362649 | 19 | 0.031 |
| CDK4 | ENSP00000257904 | 10 | 0.032 |
| HIST1H3A | ENSP00000350275 | 8 | 0.033 |
| SERPINE1 | ENSP00000223095 | 18 | 0.034 |
| IRS1 | ENSP00000304895 | 6 | 0.035 |
| FOS | ENSP00000306245 | 20 | 0.035 |
| RASA1 | ENSP00000274376 | 1 | 0.044 |
| PDGFRB | ENSP00000261799 | 2 | 0.045 |
| MAP2K1 | ENSP00000302486 | 2 | 0.045 |
| SRRT | ENSP00000314491 | 8 | 0.045 |
| IGFBP3 | ENSP00000370473 | 18 | 0.048 |
| HIST1H4A | ENSP00000352980 | 6 | 0.05 |
| MAPK8 | ENSP00000353483 | 8 | 0.054 |
| PLG | ENSP00000308938 | 18 | 0.057 |
| BUB3 | ENSP00000357858 | 18 | 0.058 |
| IGF1 | ENSP00000302665 | 18 | 0.06 |
| ANAPC1 | ENSP00000339109 | 13 | 0.061 |
| TSG101 | ENSP00000251968 | 18 | 0.065 |
| PTPN11 | ENSP00000340944 | 3 | 0.067 |
| BAD | ENSP00000309103 | 1 | 0.076 |
| IGF1R | ENSP00000268035 | 18 | 0.077 |
| STAT3 | ENSP00000264657 | 28 | 0.079 |
| CCND1 | ENSP00000227507 | 20 | 0.102 |
| CDC25B | ENSP00000245960 | 1 | 0.106 |
| TSC2 | ENSP00000219476 | 9 | 0.107 |
| KHDRBS1 | ENSP00000313829 | 16 | 0.112 |
| HNRNPK | ENSP00000365439 | 18 | 0.115 |
| BUB1B | ENSP00000287598 | 5 | 0.116 |
| PIK3CA | ENSP00000263967 | 1 | 0.13 |
| JAK2 | ENSP00000371067 | 7 | 0.135 |
| CCNE1 | ENSP00000262643 | 2 | 0.147 |
| HRAS | ENSP00000309845 | 2 | 0.151 |
| MAPK1 | ENSP00000215832 | 2 | 0.154 |
| SHC1 | ENSP00000401303 | 2 | 0.156 |
| HSPA8 | ENSP00000227378 | 8 | 0.161 |
| ERBB2 | ENSP00000269571 | 2 | 0.171 |
| RAF1 | ENSP00000251849 | 8 | 0.189 |
| EP300 | ENSP00000263253 | 16 | 0.203 |
| JUN | ENSP00000360266 | 18 | 0.225 |
| CBL | ENSP00000264033 | 42 | 0.226 |
| BCL2 | ENSP00000329623 | 1 | 0.229 |
| GRB2 | ENSP00000339007 | 11 | 0.232 |
| HIF1A | ENSP00000338018 | 19 | 0.246 |
| HSP90AA1 | ENSP00000335153 | 11 | 0.251 |
| MTOR | ENSP00000354558 | 1 | 0.252 |
| RB1 | ENSP00000267163 | 3 | 0.339 |
| SF3A2 | ENSP00000221494 | 2 | 0.383 |
| SNRPD3 | ENSP00000215829 | 1 | 0.394 |
| CLNS1A | ENSP00000263309 | 1 | 0.412 |
| PRMT5 | ENSP00000319169 | 1 | 0.414 |
| EGFR | ENSP00000275493 | 4 | 0.424 |
| WDR77 | ENSP00000235090 | 1 | 0.446 |
| U2AF2 | ENSP00000307863 | 1 | 0.462 |
| PCNA | ENSP00000368438 | 2 | 0.474 |
| ESR1 | ENSP00000206249 | 15 | 0.487 |
| TP53 | ENSP00000269305 | 24 | 0.551 |
| CCNB1 | ENSP00000256442 | 13 | 0.552 |
| SRC | ENSP00000350941 | 3 | 0.554 |
| CDK2 | ENSP00000266970 | 1 | 0.56 |
| UBC | ENSP00000344818 | 104 | 0.582 |
| AKT1 | ENSP00000270202 | 8 | 0.674 |
