# Supplementary material for: Identification of Candidate Genes Related to Inflammatory Bowel Disease Using Minimum Redundancy Maximum Relevance, Incremental Feature Selection, and the Shortest-Path Approach
Source: Biomed Res Int. 2017 Feb 14;2017:5741948. doi: 10.1155/2017/5741948 (PMC5331171; doi:10.1155/2017/5741948)
Supplement: Supplementary file 1 — The Supplementary Material consists of seven files. In detail, Supplementary Material I lists the MaxRel feature list and mRMR feature list obtained by mRMR method; Supplementary Material II lists the total prediction accuracy and accuracies for three classes obtained by the IFS method; Supplementary Material III lists 107 shortest path genes and their betweenness and permutation FDRs; Supplementary Material IV lists 57 candidate genes and their maximum interaction scores; Supplementary Material V lists the analysis results of DAVID on candidate genes; Supplementary Material VI lists 77 validated IBD-related genes reported in a paper; Supplementary Material VII lists the results yielded by DisGeNET. [file 5741948.f1.zip › Supp-IV.docx]

**Supplementary Material IV.** 57 candidate genes and their maximum interaction scores

| **Gene symbol** | **Ensembl ID** | **Betweenness** | **Permutation FDR** | **Maximum interaction score** |
| --- | --- | --- | --- | --- |
| ZAP70 | ENSP00000264972 | 16 | 0.016 | 999 |
| LCK | ENSP00000337825 | 21 | 0.02 | 999 |
| CNOT1 | ENSP00000320949 | 6 | 0.004 | 998 |
| HCFC1 | ENSP00000309555 | 36 | <0.001 | 997 |
| CNOT4 | ENSP00000354673 | 6 | 0.008 | 987 |
| S100A6 | ENSP00000357708 | 19 | <0.001 | 987 |
| STK11 | ENSP00000324856 | 19 | <0.001 | 986 |
| FASLG | ENSP00000356694 | 19 | <0.001 | 985 |
| THBD | ENSP00000366307 | 19 | <0.001 | 985 |
| BTG1 | ENSP00000256015 | 13 | 0.006 | 981 |
| TLR4 | ENSP00000363089 | 19 | <0.001 | 970 |
| HDAC1 | ENSP00000362649 | 19 | 0.031 | 967 |
| YWHAZ | ENSP00000309503 | 19 | 0.009 | 962 |
| F2 | ENSP00000308541 | 19 | <0.001 | 953 |
| FOS | ENSP00000306245 | 20 | 0.035 | 950 |
| TRAK1 | ENSP00000328998 | 19 | <0.001 | 946 |
| SERPINE1 | ENSP00000223095 | 18 | 0.034 | 933 |
| PLCG1 | ENSP00000244007 | 19 | 0.022 | 927 |
| RUNX1 | ENSP00000300305 | 19 | <0.001 | 927 |
| VEGFC | ENSP00000280193 | 19 | <0.001 | 919 |
| NNAT | ENSP00000062104 | 19 | <0.001 | 889 |
| PLAT | ENSP00000220809 | 18 | 0.017 | 888 |
| LEPR | ENSP00000330393 | 19 | <0.001 | 868 |
| IL6 | ENSP00000258743 | 19 | 0.002 | 855 |
| VEGFA | ENSP00000361125 | 19 | 0.008 | 826 |
| FGFR1 | ENSP00000380280 | 19 | 0.003 | 801 |
| PDPN | ENSP00000294489 | 19 | <0.001 | 778 |
| HIST2H2BE | ENSP00000358151 | 19 | <0.001 | 755 |
| FGF3 | ENSP00000334122 | 19 | <0.001 | 730 |
| LUC7L3 | ENSP00000240304 | 19 | <0.001 | 717 |
| MEN1 | ENSP00000337088 | 28 | 0.005 | 675 |
| ANXA1 | ENSP00000257497 | 1 | 0.007 | 641 |
| ELL | ENSP00000262809 | 18 | 0.002 | 641 |
| SRSF1 | ENSP00000258962 | 19 | 0.001 | 639 |
| CDC37 | ENSP00000222005 | 19 | 0.003 | 619 |
| FADD | ENSP00000301838 | 19 | <0.001 | 433 |
| IRS1 | ENSP00000304895 | 6 | 0.035 | 393 |
| GP1BA | ENSP00000329380 | 19 | 0.003 | 374 |
| CDK4 | ENSP00000257904 | 10 | 0.032 | 369 |
| ANXA2 | ENSP00000346032 | 18 | 0.01 | 364 |
| KDR | ENSP00000263923 | 19 | 0.001 | 333 |
| IRAK1 | ENSP00000358997 | 19 | 0.002 | 317 |
| IL6ST | ENSP00000338799 | 2 | 0.029 | 303 |
| PDGFRB | ENSP00000261799 | 2 | 0.045 | 297 |
| RIPK1 | ENSP00000259808 | 19 | 0.003 | 271 |
| JUND | ENSP00000252818 | 28 | 0.005 | 263 |
| RASA1 | ENSP00000274376 | 1 | 0.044 | 241 |
| IGFBP3 | ENSP00000370473 | 18 | 0.048 | 241 |
| MAP2K1 | ENSP00000302486 | 2 | 0.045 | 221 |
| HIST1H3A | ENSP00000350275 | 8 | 0.033 | 161 |
| HNRNPA0 | ENSP00000316042 | 17 | 0.002 | 160 |
| SNF8 | ENSP00000290330 | 18 | 0.006 | 0 |
| SRRT | ENSP00000314491 | 8 | 0.045 | 0 |
| WDR5 | ENSP00000351446 | 36 | <0.001 | 0 |
| VPS28 | ENSP00000366565 | 18 | 0.019 | 0 |
| VPS36 | ENSP00000367299 | 18 | 0.015 | 0 |
| UBE2D2 | ENSP00000381717 | 6 | 0.011 | 0 |
