# Supplementary material for: Identification of Candidate Genes Related to Inflammatory Bowel Disease Using Minimum Redundancy Maximum Relevance, Incremental Feature Selection, and the Shortest-Path Approach
Source: Biomed Res Int. 2017 Feb 14;2017:5741948. doi: 10.1155/2017/5741948 (PMC5331171; doi:10.1155/2017/5741948)
Supplement: Supplementary file 1 — The Supplementary Material consists of seven files. In detail, Supplementary Material I lists the MaxRel feature list and mRMR feature list obtained by mRMR method; Supplementary Material II lists the total prediction accuracy and accuracies for three classes obtained by the IFS method; Supplementary Material III lists 107 shortest path genes and their betweenness and permutation FDRs; Supplementary Material IV lists 57 candidate genes and their maximum interaction scores; Supplementary Material V lists the analysis results of DAVID on candidate genes; Supplementary Material VI lists 77 validated IBD-related genes reported in a paper; Supplementary Material VII lists the results yielded by DisGeNET. [file 5741948.f1.zip › Supp-VI.docx]

**Supplementary Material VI.** 77 validated IBD-related genes reported in a paper

ATG16L2

C13orf31

CARD8

CCR1

CCR3

CCR6

CD302

CHADL

CSDC2

CTC-347C20.2

CYTH1

DOCK7

DUSP5

ELF1

FCGR2A

FCHO2

FCHSD2

FGFR1OP

FOXD1

HLA

HLA-DOB

HLA-DQA1

HLA-DQA2

HLA-DQB1

HLA-DRB1

ICOSLG

IL10

IL12B

IL21

IL23R

INSL4

INSL6

IRF8

JAK2

KLF3

L3MBTL2

LOC100129633

LOC645266

microRNA2276

MIR3939

MYO9B

NFATC1

NFKBIZ

NHP2L1

NKX2-3

NOD2

NoGene

OTUD3

PHF5A

PLA2G2E

PLCL1

PMM1

PRKAB1

PSMB9

PTGS2

REL

RNASET2

RNF186

RP11-525A16.1

RUNX3

SLC25A15

SLC26A3

SMNDC1

SOX11

STARD10

STAT3

TBC1D1

TEF

TMEM171

TMEM174

TNFSF15

TNFSF8

TRIM8

USP1

USP36

VAMP3

WBP4
